# Improvement of thrombosis management in patients with cancer: a practical consensus document of recommendations for cancer-associated thrombosis patients’ healthcare in Spain

**DOI:** 10.1007/s12094-023-03379-z

**Published:** 2024-02-13

**Authors:** Andrés Muñoz Martín, Antonio Javier Trujillo-Santos, Edelmira Martí, Luis Jara-Palomares, Raquel Macías Montero, Enrique Gallardo, Juan José López-Núñez, Elena Brozos-Vázquez, Verónica Robles-Marinas, Pedro Pérez-Segura, Pedro Ruíz-Artacho

**Affiliations:** 1 Oncology Department, Hospital General Universitario Gregorio Marañón, Universidad Complutense Madrid, Madrid, Spain; 2 Internal Medicine Department, Hospital General Universitario Santa Lucía, Cartagena, Murcia Spain; 3 Haematology Department, Hospital Clínico Universitario de Valencia, Valencia, Spain; 4 Pneumology Department, Hospital Universitario Virgen del Rocío, Seville, Spain; 5 CIBER de Enfermedades Respiratorias (CIBERES), Instituto de Salud Carlos III, Madrid, Spain; 6 Oncology Department, Hospital de Badajoz, Badajoz, Spain; 7 Oncology Department, Parc Taulí Hospital Universitari, Barcelona, Spain; 8 Internal Medicine Department, Hospital Germans Trias i Pujol, Barcelona, Spain; 9 Oncology Department, Hospital Clínico Universitario de Santiago, A Coruña, Spain; 10 Hematology Department, Hospital Universitario de Cabueñes, Gijón, Spain; 11 Oncology Department, Hospital Clínico Universitario San Carlos, Madrid, Spain; 12 Internal Medicine Department, Clínica Universitaria Navarra, Madrid, Spain

**Keywords:** Cancer-associated thrombosis, Thrombosis management, Practical consensus, Spain

## Abstract

Cancer patients are at risk of venous thromboembolism (VTE), its recurrence, but also at risk of bleeding while anticoagulated. In addition, cancer therapies have been associated to increased VTE risk. Guidelines for VTE treatment in cancer patients recommend low molecular weight heparins (LMWH) or direct oral anticoagulants (DOAC) for the initial treatment, DOAC for VTE short-term treatment, and LMWH or DOAC for VTE long-term treatment. This consensus article arises from a collaboration between different Spanish experts on cancer-associated thrombosis. It aims to reach an agreement on a practical document of recommendations for action allowing the healthcare homogenization of cancer-associated thrombosis (CAT) patients in Spain considering not only what is known about VTE management in cancer patients but also what is done in Spanish hospitals in the clinical practice. The text summarizes the current knowledge and available evidence on the subject in Spain and provides a series of practical recommendations for CAT management and treatment algorithms to help clinicians to manage CAT over time.

**Supplementary Information:**

The online version contains supplementary material available at 10.1007/s12094-023-03379-z.

## Introduction

The association of cancer with an increased risk of venous thromboembolism (VTE), including deep vein thrombosis (DVT) and pulmonary embolism (PE), has long been known [1, 2]. Cancer patients are at risk of VTE recurrence, but also at risk of bleeding while anticoagulated [3]. Finally, cancer therapies also have been associated to increased risk of VTE [4, 5]. Anticoagulant therapy with low-molecular-weight heparin (LMWH) has been the preferred approach recommended by clinical practice guidelines during years but recently several guidelines indicated direct oral anticoagulants (DOAC) as another option for VTE treatment in many, but not all, cancer patients [6–10].

Several studies show that direct oral anticoagulants (DOAC) are a convenient and effective treatment alternative to low molecular weight heparins (LMWH) in patients with VTE [11–13]. More recently, DOAC has been compared to LMWH in randomized clinical trials in patients with VTE and cancer, where apixaban, edoxaban, and rivaroxaban showed to be non-inferior to dalteparin in preventing VTE recurrence [14–16]. SELECT- D and Hokusai clinical trials showed that rivaroxaban and edoxaban, respectively, were associated with lower VTE recurrence vs LMWH [14, 15]. In the same line, in the Caravaggio study, apixaban showed to be non-inferior to LMWH in preventing VTE recurrence [16]. Regarding bleeding, the Caravaggio study [16] showed similar frequencies of major bleeding with apixaban and dalteparin (including gastrointestinal bleeding), results in contrast to SELECT-D and Hokusai trials where a higher bleeding incidences with other direct oral anticoagulants compared to dalteparin were found [14, 15]. Regarding clinically relevant nonmajor bleeding (CRNMB), these were numerically higher for apixaban compared to dalteparin in the Caravaggio study [17], mainly due to bleeding into genitourinary system and upper airways, but results were in line with other previous studies [14, 15].

Guidelines for VTE treatment in patients with cancer recommend LMWH or DOAC for the initial treatment, DOAC for VTE short-term treatment, and LMWH or DOAC for VTE long-term treatment [8]. At this point, it is important to consider the burden associated with daily subcutaneous injections of LMWH, and, thus, the potential low adherence when considering long-term treatment [17]. In this regard, DOAC therapy may provide a convenient and effective alternative to LMWH. A cost-effectiveness analysis between LMWH and DOAC (but not including all DOACs) showed potential benefit of DOAC vs LMWH [18]. In addition, a recent study has demonstrated that treatment with DOAC is more cost-effective and cost-saving for VTE treatment than LMWH from the Spanish healthcare system perspective [19].

The main objective of this Spanish expert’s meeting was to reach an agreement on a practical document of recommendations for action allowing the healthcare homogenization of cancer-associated thrombosis (CAT) patients in Spain considering not only what is known about VTE management in cancer patients but also what is done in Spanish hospitals in the clinical practice.

### Management of the oncological patient with VTE

In the healthcare route, several hospital services/departments are involved in the different steps (diagnosis, treatment, and follow-up [extended treatment]) of CAT management (Table 1). In addition, if the patient has nonvalvular atrial fibrillation (NVAF), cardiology, oncology, haematology departments (cardio-onco-haematology process) are also involved during the follow-up stage. Spanish experts agreed that a great heterogeneity was observed related to the care of cancer patients with VTE according to the hospital where they are treated, both in diagnosis and treatment (first 6 months) stages, and extended treatment (> 6 months) stage.

**Table 1:**
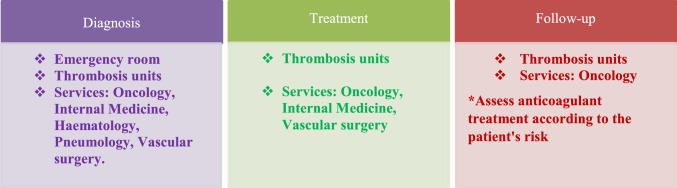
Hospital departments involved in diagnosis, treatment, and follow-up steps

**Fig. 1:**
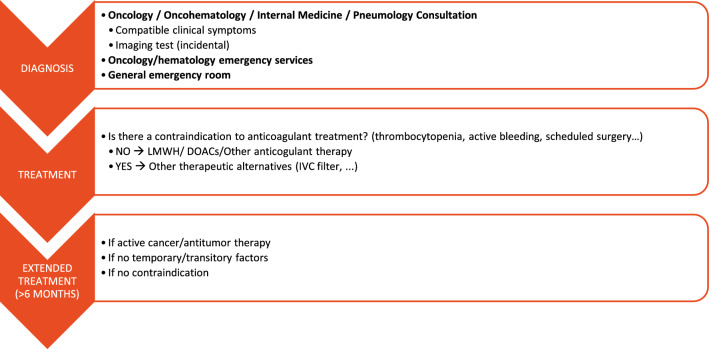
CAT patient healthcare route

Diagnosis is usually made in outpatient services/settings (oncology, internal medicine, pneumology and/or haematology). At treatment stage, therapy is usually established based on whether there is a contraindication to anticoagulant therapy whereas for extended treatment, beyond 6 months, this is usually prolonged if there is active cancer or antitumor therapy and there are no other factors contraindicating treatment continuation. Active cancer is considered a risk factor for VTE recurrence. Figure 1 shows a summary of healthcare route for CAT patients.

**Fig. 2:**
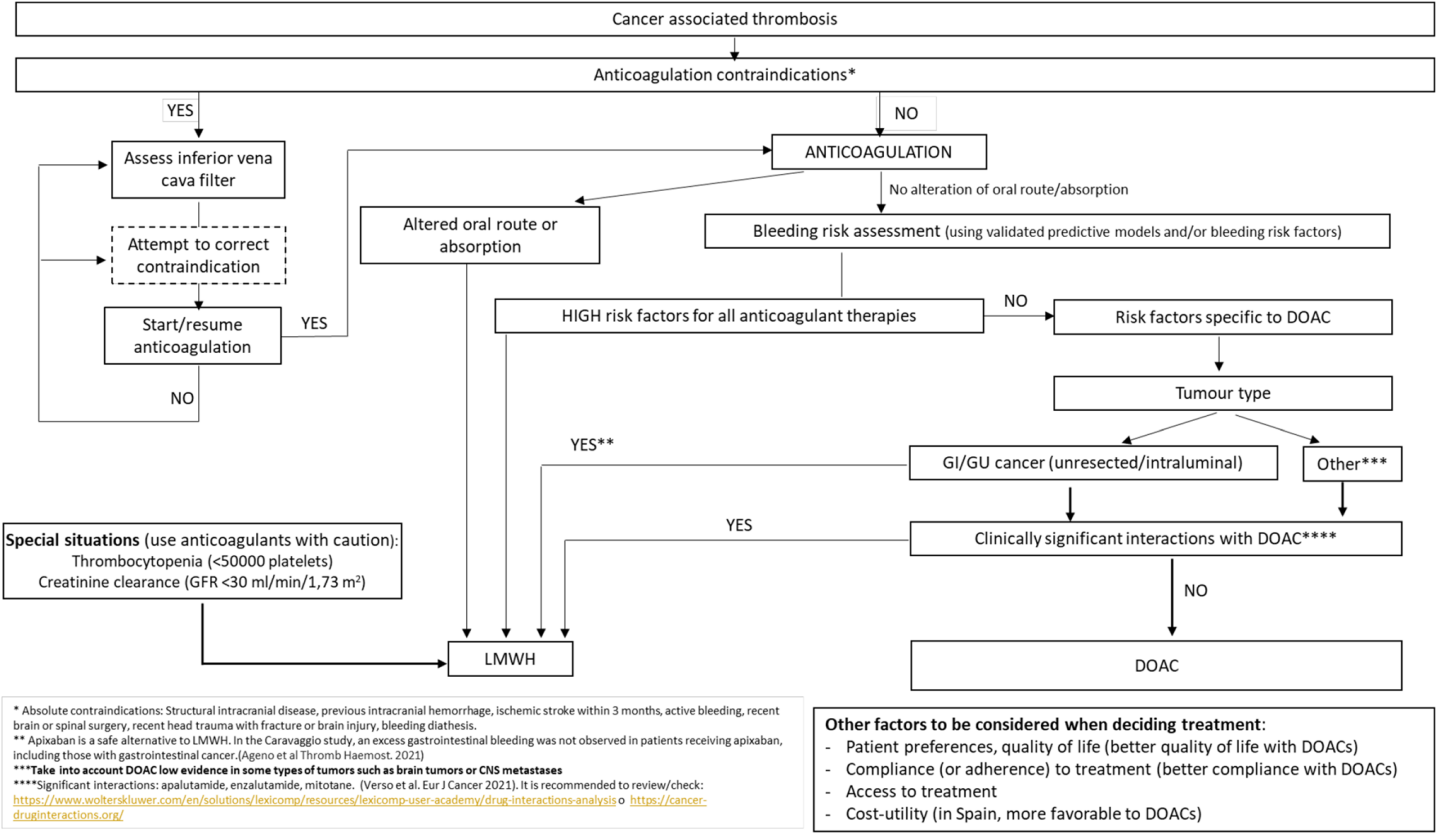
Risk stratification algorithm for anticoagulant therapy in patients with cancer-associated thrombosis. *DOAC* Direct oral anticoagulants; *GFR* glomerular filtration rate; *GI* gastrointestinal; *GU* genitourinary; *LMWH* low molecular weight heparin. *Absolute contraindications: active major bleeding, major surgery in the last 24 h, severe liver and kidney disease, severe uncontrolled hypertension, haemorrhagic retinopathy. **Apixaban is a safe alternative to LMWH. In the Caravaggio study, an excess gastrointestinal bleeding was not observed in patients receiving apixaban, including those with gastrointestinal cancer (Ageno et al. Thromb Haemost. 2021). ***Take into account DOAC low evidence in some types of tumours such as brain tumours or CNS metastases. ****Significant interactions: apalutamide, enzalutamide, mitotane. (Verso et al. Eur J Cancer 2021). It is recommended to review/check: https://www.wolterskluwer.com/en/solutions/lexicomp/resources/lexicomp-user-academy/drug-interactions-analysis o https://cancer-druginteractions.org/

**Fig. 3:**
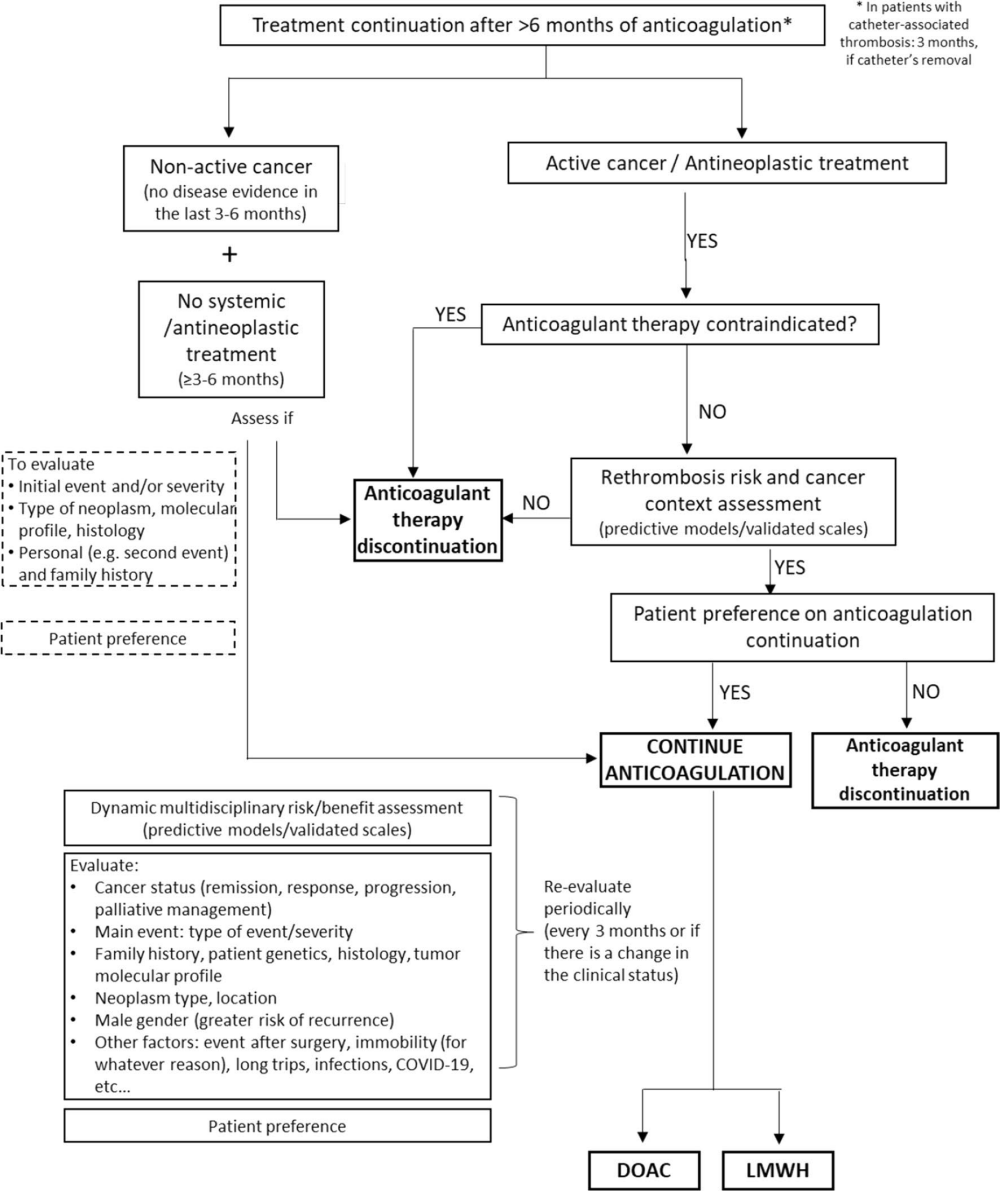
Treatment continuation algorithm beyond 6 months. *DOAC* Direct oral anticoagulants; *LMWH* low molecular weight heparin

**Fig. 4:**
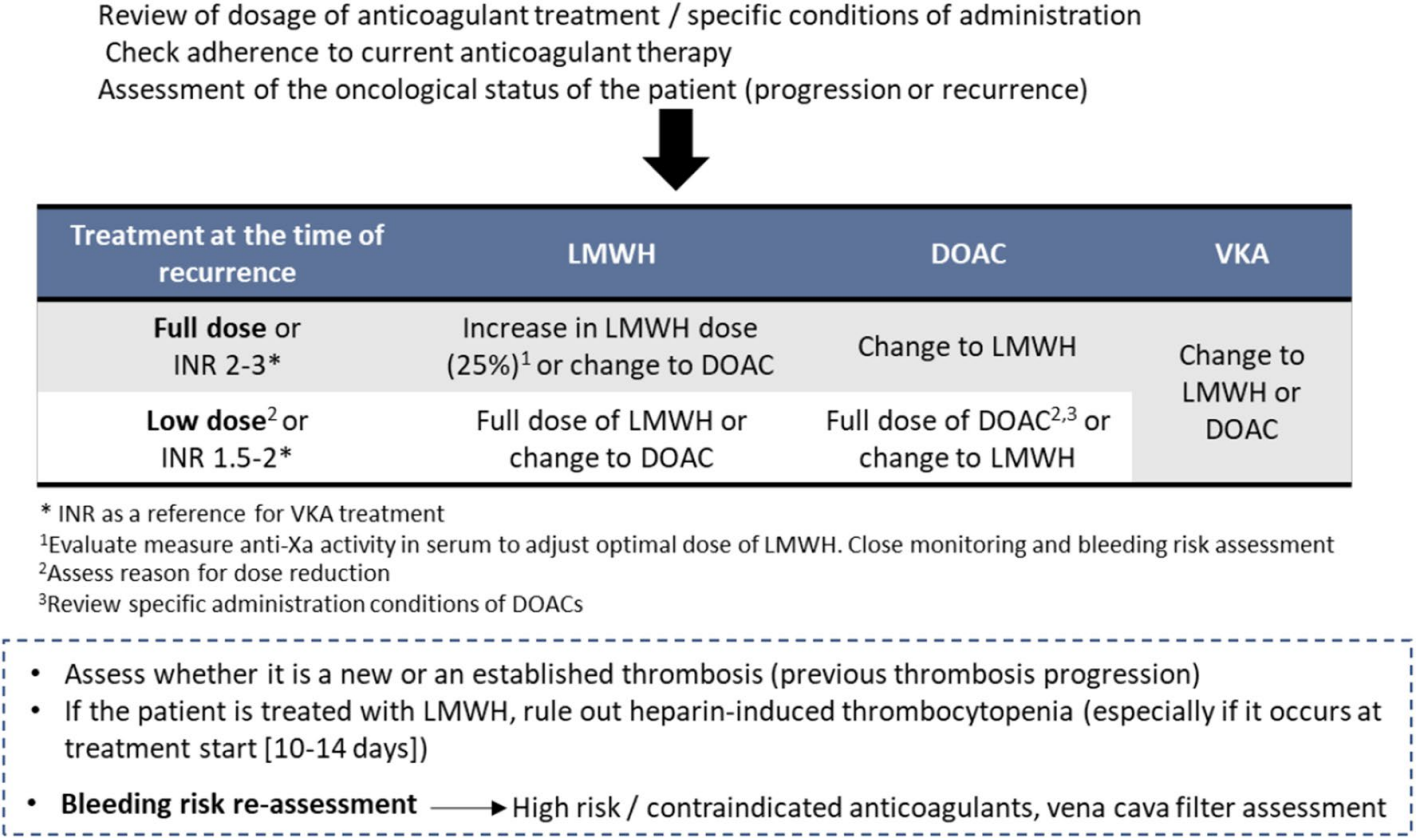
Algorithm for VTE recurrence during anticoagulation. *DOAC* Direct oral anticoagulants; *INR* international normalized ratio; *LMWH* low molecular weight heparin; *VKA* vitamin K antagonist

The Spanish multidisciplinary expert’s meeting, which included 11 physicians with expertise in medical oncology, internal medicine, pneumology and haematology, discussed on anticoagulant therapy in patients with CAT at diagnosis and CAT treatment beyond 6 months, as well as management of VTE recurrence during anticoagulant therapy. At the end of the meeting, the group of experts achieved a consensus on possible CAT treatment algorithms at the diagnosis step (Fig. 2), after 6 months of anticoagulant therapy (Fig. 3), and during VTE recurrence (Fig. 4).

#### The existence of thrombosis units and their utility

All experts brought their experience in the management of cancer patients with VTE in their hospitals. Most of them considered that the existence of thrombosis units is useful/very useful since CAT is a complex multidisciplinary pathology, although not all hospitals have a Thrombosis Unit in the centre.

In hospitals without thrombosis unit, the different services involved cooperate by bringing into communication with one another to help diagnosis/treatment of patients. In the other side, experts from hospitals with thrombosis units report that, depending on the hospital, these units depend on the internal medicine service (in most of cases), pneumology or haematology unit. In some hospitals, a specific consultation/Thrombosis Unit has been created with medical oncology specialists in charge within the medical oncology service. Thrombosis Units not only take care of patients with thrombosis, but they also act as interconsultants in case the patient is hospitalized for any other reason.

In general, diagnosis is usually carried out by vascular surgery and radiology services; while the medical oncology service itself, with the haematology/pneumology/internal medicine services (in case of a joint evaluation is needed) oversee the treatment of CAT patient.

There are also hospitals that have a multidisciplinary thrombosis commission with monthly meetings to discuss the specific clinical cases and with the purpose of generating protocols or guides for the hospital itself.

At this point, it should be noted the importance of the nursing service, which can help to educate the patient in CAT/thrombosis and to provide support with the medication. In this sense, experts suggested that it would be important to prepare a document addressed to oncology nurses to educate about the handling of these patients.

As a conclusion, the three main points emerged were: when there is a Thrombosis Unit or any structured Unit dealing with CAT patient management, teams for the management of these patients with CAT should be multidisciplinary; patient’s medical care should consider both active cancer and thrombosis; and importance of the existence of a Thrombosis Units (with a multidisciplinary team).

#### Adequate management of the CAT patient: should there be indicators of quality, safety, follow-up, and adherence? Which ones?

All the experts agreed that there must be indicators to be able to determine if CAT patient management is appropriate, although this is not often done in the clinical practice. An important point for management is adherence to treatment. Experts also discussed quality indicators for evaluating patient management: e.g. to follow-up the progress or how the patient perceives the treatment; and stated that it is important to consider readmissions, re-thrombosis, bleeding, the collection of patient reports (Patient Reported Outcomes [PROs]) and patient experience with anticoagulant therapy:

- The ideal would be to collect the PROs in the medical history.
- Consultations can be used to collect questionnaires on quality of life and patient satisfaction through surveys.
- The importance of the involvement of patient associations/organizations or associations of patients with thrombosis is highlighted. These organizations could take the advantage of to promote and give visibility to the patient with CAT and raise awareness among patients to improve treatment adherence.

#### Medical specialties and connection with other specialists/services/hospitals

Regarding the methods and criteria of relationship with other specialists/hospitals and VTE consultation, it should be noted that there are different ways to contact the several services/departments involved in CAT management as each hospital has its own communication and relationship system between the different specialists involved. In addition, although the delay time per patient varies by hospital, a prompt response is guaranteed (less than 24 h in most cases).

In the specific case of CAT patients, depending on the hospital and admission route (oncology/vascular/emergency room…), the procedure varies. In some cases, CAT is handled as a specific consultation in the VTE unit and is attended on ward or in consultation by internal medicine. In other cases, if nursing or physicians suspect of CAT, the latter will refer the patient to diagnostic services (vascular/radiology) or to the emergency room (diagnosis on the same day or in 24 h) and treatment will be started on the same day.

For those experts reporting information on follow-up/visits, the procedure also varies depending on the hospital. In some hospitals, e.g., CAT patient visits tend to be prioritized over non-cancer patients, since these patients tend to have more complications, have added morbidity but also due to the disease burden of cancer itself. In other centres, appointments/visits for CAT patient are the same as for patients with a thrombotic event of other characteristics.

#### Risk factors’ assessment

Regarding risk factors’ assessment and their relevance to classify patients at high risk, the experts provide the most relevant risk factors according to their professional experience. Most experts agree that thrombotic burden, as well as bleeding/bleeding risk factors, type and stage of tumour, and oncological situation (progression, response, or remission) are the most principal factors to assess the patient and determine the risk the patient is exposed to. Other factors that can be considered secondary/at the second level are comorbidities, or the patient’s own characteristics (i.e. age, socioeconomic factors…etc.).

Establish a checklist and/or risk predictive models to assess the main risk factors to facilitate decision-making (It may be completed by nurses).

#### Barriers

The main barriers discussed were related to lack of training/education of the professionals involved in CAT management, the use and evaluation through PROs, and proper communication with patients.

The five main barriers described were:

- *Awareness*: Education and training for professionals, awareness of both bleeding risk and thrombotic risk, patient education on the warning signs of thrombosis.
- *Develop protocols*: The needs to have protocols and to be uniform among the different hospitals managing CAT patients was stressed. Regarding protocols, it was also discussed the importance of preparing guidelines considering these protocols to be able to carry out a continuous assessment of the follow-up in everyday clinical practice /hospitals.
- *Diagnosis*: Perform lower limb ultrasound during hospitalization. Possibility to perform lower limb ultrasound in consultation in some centres.
- *Funding*: DOACs are only used in those patients who obtain funding (very few) or in those who can afford them.
- *Treatment*: Important to identify the interactions between anticoagulant therapy and anticancer drugs to adapt the best anticoagulant treatment to each patient. Therefore, better tools to assess risk of interactions are needed as current available risk tools present a great heterogeneity in the information provided.

### Recommendations for improvements nationwide

According to experts, in most cases, the patient arrives at the emergency room where he/she can stay for several hours until the tests needed are performed. Experts also stated that, when CAT is suspected, it is important to perform the same tests for all patients and to be done as soon as possible for proper diagnosis and treatment. Therefore, it would be good to reach a consensus on what tests should be performed in the emergency room at diagnosis. In terms of follow-up of the patient with a VTE diagnosis, it should be noted that, in the process of patient diagnosis/monitoring, oncology usually only takes care of the follow-up if patient has a cancer diagnosis. If there is no cancer diagnosis, other hospital services/departments take care of the patient follow-up.

In summary:

- There is high heterogeneity and variability between hospitals in the diagnostic healthcare route for patients with cancer and thrombosis.
- In terms of hospital departments/services involved (medical oncology, haematology, pneumology, internal medicine), the follow-up of patients with cancer and thrombosis is also very heterogeneous in the different Spanish hospitals. Multidisciplinary management would be good throughout the whole process of cancer-associated venous thromboembolic disease. In doubtful and/or complex cases, these services are consulted for joint decision-making.

#### Key points for improvement of the healthcare route

1. Include a CAT management protocol within the healthcare route, monitor it at the end of the year, with impact in the program contract with the hospital manager and the corresponding regional Health Service. This would help the engagement of professionals and to assess the evolution of CAT annually. Continuous evaluation with quality parameters.
2. Multidisciplinary involvement to be agreed according to the hospital/area: Oncology, internal medicine, haematology, pneumology, vascular surgery, emergency, primary care, nursing.
3. Primary care: It is important to educate CAT patient in thrombosis to avoid admission to the emergency room. A not small percentage of the cases could be initially diagnosed and treated in primary care and only some should be admitted. After initial diagnosis and treatment, follow-up could be performed by oncology/hematology/pneumology/internal Medicine. In this regard, the SEC (from the Spanish acronym: Spanish Society of Cardiology: Sociedad Española de Cardiologia) project (SEC-PRIMARIA, cardiology and primary care integrating project, which aims to improve continuous care, training and communication between professionals at different healthcare levels in the field of cardiovascular diseases [20]) should be taken as an example to follow. For this reason, the SEC has published a monograph addressed to the cardio-onco-haematology consultation for the management of antineoplastic toxicities with objectives that can be adapted to patients with CAT. Among others, this monograph discusses on the importance of the healthcare route for cancer patients and the need for cooperation and consensus between the different healthcare levels to:
  Reduce cardiovascular complications from onco-haematological treatments, promoting baseline risk stratification and optimal management of classic cardiovascular risk factors during the cancer process.
  Facilitate antitumor therapy and minimize its interruptions.
  Early identification of any cardiovascular complication to initiate appropriate treatment at early stage.
    Improve the prognosis of long-term cancer survivors.
  Improve the prognosis of long-term cancer survivors.
4. Improve patient follow-up since it is not well defined (how to do it, until when… etc.). It is important to clearly define the most appropriate follow-up for the patient (different monitoring schemes depending on the CAT patient profile):
  In high-risk, chronic patient: part of the follow-up could be done by phone/video call (regular checks). This would be possible in some patient profiles, for example, in patients who have stable disease. In addition, to minimize patient discomfort, thrombosis consultation can be match up with another patient visit to the hospital.
  Specify the treatments offered to the patient and the treatment chosen (reasons for accepting or, specially, rejecting a treatment).
  It is recommended to carry out quality of life questionnaires.
  Experts also recommended to carry out PRO–PROMs.

#### Points for improvement in the definition of active cancer, high-risk factors, medical history information

1. Have a tool for prevention and detection of patients at risk (Checklist of healthcare route for Oncology) and define detection methods of high-risk patients. On this point, the role of nursing and electronic medical record alerts are important.
2. Establish quality indicators.
3. Description of the event in the medical history.
4. Ease the results’ analysis.
5. Define what is active cancer.

#### Points for training improvement

1. Involve all professionals by educating/training, sensitizing, and raising awareness about the treatment and follow-up of CAT patient, by doing training courses for the professionals implicated in the management of CAT patient. For medical oncology residents, thrombosis associated with cancer is included in the 5-year residency training curriculum. Thus, the specialist in Medical Oncology at the end of his/her residency should know how to handle CAT (Master SEOM includes specific CAT training).
2. Education and training of patients (involvement of oncology nursing staff).

#### Key points to consider in tests and diagnosis

After bringing their own experience in the different hospitals, experts discussed points of improvements related to tests and CAT diagnosis. Among them, the following ones were highlighted:

1. Organize multidisciplinary work units involving nursing to manage CAT patients in the best possible way (in each hospital).
2. Have a precise definition of active cancer.
3. Collect family medical history, not just the patient medical history.
4. Collect/review the medical records (scores/medical history) periodically as it is a dynamic process.
5. Establish a checklist with risk scores to help assessment (e.g., for nursing)
6. To have available specific predictive models of cancer for diagnosis and treatment.
7. Importance of analysing the D-dimer, especially in patients with a low-risk of recurrence. D-dimer determination helps in patient management and treatment, but it has not been adequately studied in the literature or standardized in clinical practice.
8. Train nursing and clinical oncologists in CAT patient management (e.g., SEOM has started a collaboration with SEEO [Spanish Society of Oncology Nursing] to involve and train oncology nursing staff in CAT).
9. Know cancer stage and/or progression to determine treatment (therapeutic or prophylactic dose)/anticoagulant therapy continuation (close relationship between cancer progression and thrombosis risk, and recurrence and bleeding risk).

A summary of the main points for improvements regarding tests and diagnosis of CAT is shown in Supplementary Table 1.

After discussing all prior key points, the different experts provided recommendations concerning four main areas (protocol/consensus documents, DOACs, training/education, and other issues) (Table 2).

**Table 2:** Recommendations to implement improvements in Spain

Protocols, consensus documents	DOAC	Training/education	Other
Single national protocol^a^	Patient with thrombocytopenia: establish DOAC dose when platelets < 50,000	Educational programs of multidisciplinary VTE units’ network in patients with CAT^b^	Treatments, dosage and adherence to treatment audits
Joint national register	DOAC endorsement	Education/awareness of cancer patients through conferences with patients	Similar/same additional tests throughout the whole national territory
Protocol for management of complex or special situations^c^	Update the therapeutic positioning reports (Informes de Posicionamiento Terapéutico [IPT]) of DOACs to be able to be prescribed in all the autonomous communities without restrictions	Training: sessions, courses and talks for healthcare professionals involved in the diagnosis, monitoring and treatment of cancer patients (with and without thrombosis)	Access to the same drug therapy (i.e., DOAC) in the different autonomous communities
Preparation of consensus documents/clinical practice guidelines^d^		Social media campaigns aimed at patients	Facilitate access in case of suspicion and in patients with CAT: teleconsultation, applications, follow-up, etc
			Promote both independent and pharmaceutical industry clinical research with trials answering questions in specific clinical scenarios and/or multicentre prospective studies in cancer patients
			Promote multidisciplinary VTE committees and/or commissions and monographic consultations on CAT
			Promote the formation of oncothrombosis units

*CAT* Cancer associated thrombosis; *DOAC* direct oral anticoagulants; *VTE* venous thrombosis embolism

^a^Including the implication that each medical specialty must carry out, as well as periods/when the patient must be reassessed and treated. Management protocols must include diagnosis, therapy, follow-up…

^b^Composed of hospital specialists in charge of assessing these patients: internal medicine, medical oncology, haematology, radiation oncology, general and digestive surgery, other surgeries, radiodiagnosis, vascular surgery

^c^It would allow prospective studies to validate protocols, as well as their improvement

^d^Through regular meetings with experts. Consensus document on the CAT approach. The different medical specialties involved in consensus document will allow to obtain a global vision of the patient, not only considering the cancer disease and drug interactions

## Conclusion

CAT is a disease encountered increasingly, with an important impact on patients’ life. Its management can be ameliorated, with different areas of potential significant improvement. Besides, anticoagulant therapy in CAT patients should be individualized considering several factors, among them, bleeding risk, type and stage of tumour, and oncological situation. For these reasons, we provide some practical recommendations for CAT management and treatment algorithms to help clinicians to manage CAT over time.

### Supplementary Information

Below is the link to the electronic supplementary material.

Supplementary file1 (DOCX 18 KB)
