# Supplementary material for: Improvement of thrombosis management in patients with cancer: a practical consensus document of recommendations for cancer-associated thrombosis patients’ healthcare in Spain
Source: Clin Transl Oncol. 2024 Feb 13;26(6):1319–28. doi: 10.1007/s12094-023-03379-z (PMC11108917; doi:10.1007/s12094-023-03379-z)
Supplement: Supplementary file 1 — Supplementary file1 (DOCX 18 KB) [file 12094_2023_3379_MOESM1_ESM.docx]

# **Supplementary table**

**Supplementary Table** 1. Key points for improvement in tests and CAT diagnosis.

|  | **POINTS FOR IMPROVEMENT** |
| --- | --- |
| **Medical history** | Collect always previous history of thrombosis (from the patient and relatives). Family history of VTE/thrombosis. Assess symptoms and/or signs in incidental VTE.  Increase time for assessment.  Perform assessment of the family history/risk factors of CAT at diagnosis and periodically (since it is a dynamic process). |
| **Laboratory criteria** | More time for assessment.  Petitions based on probability.  Record D-dimer determination. |
| **Radiological criteria** | Availability and/or facility to comment on the examinations with Radiology.  Improve communication with Radiology. |
| **Clinical criteria** | Nursing consultation.  Establish a clear definition of active cancer.  Educate to improve the index of suspicion.  Inform the patient about the alarm symptoms periodically; thrombotic risk with cancer, and haemorrhagic risk with treatment. |
| **Criteria for choosing anticoagulant treatment** | Joint decision-making between doctor and patient.  Improve knowledge about DOACs (including drug-drug interactions*) by the rest of the services, especially in Oncology.  Establish or define the best sources to check drug-drug interactions.  DOAC funding.  Coordination between services/departments. |
| **Long-term follow-up** | Have a specific thrombosis consultation to assess CAT patients allowing synchronous follow-up by Oncology and the VTE unit.  Involvement of all related services.  More time for evaluation.  Improvement of circuits with primary care and involvement of primary care physicians. |
| **Long-term treatment** | DOAC funding  More time for patient and appropriate treatment assessment.  Improve communication with primary care.  Establish multidisciplinary commissions.  State in the patient’s medical history if he/she meets the criteria for anticoagulant therapy discontinuation.  Consult nursing. |

* To check drug-drug interactions:

<https://www.wolterskluwer.com/en/solutions/lexicomp/resources/lexicomp-user-academy/drug-interactions-analysis>

<https://cancer-druginteractions.org/>
